# Alterations in Oral [1-^14^C] 18:1n-9 Distribution in Lean Wild-Type and Genetically Obese (*ob/ob*) Mice

**DOI:** 10.1371/journal.pone.0122028

**Published:** 2015-03-31

**Authors:** Xinxia Wang, Jie Feng, Caihua Yu, Qingwu W. Shen, Yizhen Wang

**Affiliations:** 1 College of Animal Sciences, Zhejiang University, Key Laboratory of Molecular Animal Nutrition, Ministry of Education, Key Laboratory of Animal Nutrition & Feed Sciences, Ministry of Agriculture, Zhejiang Provincial Laboratory of Feed and Animal Nutrition, Hangzhou, Zhejiang, P. R. China; 2 Department of Animal Science, Northwest A&F University, Yangling, Shaanxi P. R. China; The Ohio State University, UNITED STATES

## Abstract

Obesity may result from altered fatty acid (FA) disposal. Altered FA distribution in obese individuals is poorly understood. Lean wild-type C57BL/6J and obese C57BL/6J^*ob/ob*^ mice received an oral dose of [1-^14^C]18:1n-9 (oleic acid), and the radioactivity in tissues was evaluated at various time points. The ^14^C concentration decreased rapidly in gastrointestinal tract but gradually increased and peaked at 96 h in adipose tissue, muscle and skin in lean mice. The ^14^C concentration was constant in adipose tissue and muscle of obese mice from 4h to 168h. ^14^C-label content in adipose tissue was significantly affected by genotype, whereas muscle ^14^C-label content was affected by genotype, time and the interaction between genotype and time. There was higher total ^14^C retention (47.7%) in obese mice than in lean mice (9.0%) at 168 h (*P*<0.05). The ^14^C concentrations in the soleus and gastrocnemius muscle were higher in obese mice than in lean mice (*P*<0.05). Perirenal adipose tissue contained the highest ^14^C content in lean mice, whereas subcutaneous adipose tissue (SAT) had the highest ^14^C content and accounted for the largest proportion of total radioactivity among fat depots in obese mice. More lipid radioactivity was recovered as TAG in SAT from obese mice than from lean mice (*P*<0.05). Gene expression suggested acyl CoA binding protein and fatty acid binding protein are important for FA distribution in adipose tissue and muscle. The FA distribution in major tissues was altered in *ob/ob* mice, perhaps contributing to obesity. Understanding the disparity in FA disposal between lean and obese mice may reveal novel targets for the treatment and prevention of obesity.

## Introduction

Obesity is characterized by hyperlipidemia and elevated triglyceride stores, which may be caused in part by an alteration in the disposal of fatty acids (FAs) [1]. FAs play important roles in various cellular functions, including metabolic energy generation and storage, membrane lipid synthesis, and intracellular signaling [2]. Chronic imbalances in FA flux and metabolism cause numerous metabolic abnormalities and pathologies, including hyperlipidemia, obesity, type 2 diabetes mellitus, nonalcoholic fatty liver disease and cancer [3,4]. Alterations in FA uptake has been reported to be of primary importance for the regulation of FA disposal [5], thus understanding the changes in FA uptake and the underlying rationale in obese individuals will provide insight into the mechanism leading to disordered triglyceride storage and the final obesity. Although it is known that “lipotoxicity” due to altered FA deposition is central to the pathophysiology of animal and human disorders [6], comparative studies of FA uptake and tissue distribution between lean and obese individuals have not been previously reported.

Obesity, on the other hand, influences regional variations in adipose tissue function [7], which is related to the pathology of abnormal triglyceride storage [8]. Thus, investigation into the regional changes in adipose tissue function my help to limit the jeopardy of obesity. However, alterations in regional FA storage in various adipose tissue depots in obese individuals have not been satisfactorily documented. In addition to the greater amount of adipose tissue in obese individuals, increased triglyceride content within skeletal muscle has also been reported [9], which is likely to be related to the increased demand of type I muscle fibers for FAs [10]. But how FA uptake in various muscle fiber types differs in lean and obese individuals *in vivo* is unclear.

The C57BL/6J^*ob/ob*^ mouse is a widely used genetic model of obesity that demonstrates many of the pathophysiological alterations that are observed in obese humans [11]. A complete understanding of the roles of various tissues in FA processing cannot be obtained from humans for ethical reasons. Therefore, animal models were used in current study to completely describe FA processing in various tissues *in vivo*. Here we compared the pattern of [1-^14^C] 18:1n-9 uptake and storage in various tissues from obese (C57BL/6J^*ob/ob*^) and lean wild-type (C57BL/6J) mice. Furthermore, the variations in FA uptake and retention based on fat depot and muscle fiber type were also explored, as was the expression of FA-related genes, including fatty acid translocase (FAT/CD36) [12], fatty acid binding proteins (FABPs) [13,14], long-chain fatty acyl-CoA synthetase (ACSL) [15], acyl CoA binding protein (ACBP) [16] and fatty acid transport proteins (FATPs) [17]. The results provided data on the disparities in FA disposal between lean and obese mice, which may help identify novel targets for the treatment and prevention of obesity.

## Materials and Methods

### Animals

The Committee on Animal Care and Use and the Committee on the Ethics of Animal Experiments of Zhejiang University approved all of the animal procedures. Female lean wild-type C57BL/6J and obese C57BL/6J^*ob/ob*^ mice at 4 weeks of age were purchased from Nanjing Biomedical Research Institute of Nanjing University and were maintained in a barrier facility (12:12 h light-dark cycle) with *ad libitum* access to food and water. After one week of acclimatization, all animals (5 weeks old) were fasted overnight and given a single oral dose of 2.1 μCi of [1-^14^C] 18:1n-9 (American Radiolabeled Chemicals, St. Louis, MO, USA) per mouse. The animals continued to have *ad libitum* access to regular food and water until the biopsies were performed.

### Tissue collection

Three lean and obese mice were euthanized 0, 4, 12, 24, 48, 96 and 168 h after dosing with the isotopic tracer. Orbital blood was drawn after the mice were euthanized with carbon dioxide. Each mouse was weighed and dissected into 18 components as detailed below. The head was severed at the neck, and the brain was collected. The body was placed on ice during the dissection. The gallbladder was removed before collecting the liver and other abdominal tissue to avoid contamination with bile fluid. The major organs, including the heart, lungs, liver, pancreas, kidneys and spleen, were collected. The gastrointestinal tract (from the duodenum to the anus) was emptied and rinsed with ice-cold saline (0.85% NaCl). The stomach was subsequently removed and treated similarly. Skin without fur was collected from the carcass. Subcutaneous adipose tissue (SAT), visceral adipose tissue (VAT), perirenal adipose tissue (PAT), and mesenteric adipose tissue (MAT) as well as the soleus (SOL), gastrocnemius (GAS) and extensor digitorum longus (EDL) muscles were collected separately. The remaining carcass, containing the skull, ribs, bones, cartilage, paws, tail and the parts not listed above, was carefully washed with cold normal saline after dissection, blotted and homogenized in a grinding machine, and 1 g of the minced carcass was used in the radioactivity assay [18].

### Radiotracer detection

Tissues from each organ sample were accurately weighed and subsequently dissolved in SOLVABLE (Perkin Elmer, Waltham, MA, USA) at 50°C overnight. Serum samples (50 μl) were aliquoted into scintillation vials. Ultima Gold (5 ml) (Perkin Elmer, Waltham, MA, USA) was added to each sample, and the radioactivity levels were measured using a Multi-Purpose Scintillation Counter LS6500 (Beckman Coulter, Brea, CA, USA).

### Lipid extraction and analysis

Total lipids were extracted from adipose tissue as described by Folch et al. [19]. Certain lipid classes, such as phospholipids (PLs), free fatty acids (FFAs), diacylglycerol (DAG), triacylglycerol (TAG) and cholesterol ester (CE), were separated on a thin-layer chromatography (TLC) plate (Merck KGaA, Darmstadt, Germany) using a mixture of petroleum ether, diethyl ether and acetic acid (113:20:2, v/v/v) as the mobile phase. Lipids were visualized by dipping the plate in a solution of 3% cupric acetate and 8% phosphoric acid and then charring the plate for 5 min at 140°C in an oven. Petroleum ether, diethyl ether, acetic acid and cupric acetate were obtained from Sigma-Aldrich (St. Louis, MO, USA). Different spots corresponding to the lipid classes were scraped off into scintillation vials and counted as described above.

### RNA extraction, cDNA synthesis and quantitative real-time RT-PCR

Total RNA was extracted from adipose tissue and muscle using Trizol Reagent according to the manufacture’s instruction. Approximately 500 ng of total RNA was reverse-transcribed into cDNA using M-MuLV reverse transcriptase kit (Fermentas, EU, GlenBurnie, Maryland, USA) in a 20 μl reaction system according to the manufacturer’s protocol. Gene transcript levels were determined using a SYBR Premix ExTaq Kit (Takara Biotechnology Co. Ltd, Otsu, Shiga, Japan) in the ABI StepOnePlus Real-Time PCR System (Applied Biosystems, Foster City, CA, USA) according to the method described by Wang et al. [20]. Three commonly used reference genes (beta-actin, GADPH and APRT) were tested for stability using the GeNorm and NormFinder. Finally, GADPH met criteria of stability in the analyzed material and was used in gene expression analysis. The PCR primers were designed using the Primer premier software v5.0 (PREMIER Biosoft International, Palo Alto, CA, USA) and synthesized by Invitrogen. Efficiency of primers was checked from two fold serial dilutions of cDNA for each primer pair. Data were analyzed by using 2^-ΔΔCt^ and are referred to the control treatment using GADPH as a reference gene. The following primers were used:

GAPDH-F: 5’ TGTCAGCAATGCATCCTGCA 3’

GAPDH-R: 5’ CCGTTCAGCTCTGGGATGAC 3’

ACSL1-F: 5’ CGAGGGCGAGGTGTGT 3’

ACSL1-R: 5’ GTGTAACCAGCCGTCTTTGTC 3’

CD36-F: 5 TGGGAAGACAATCAAAAGGGAAGT’3’

CD36-R: 5’ GTCCTCGGGGTCCTGAGTTA 3’

ACBP-F: 5’ TTTCGGCATCCGTATCACCT 3’

ACBP-R: 5’ TTTGTCAAATTCAGCCTGAGACA 3’

FABP3-F: 5’ CCCCTCAGCTCAGCACCAT 3’

FABP3-R: 5’ CAGAAAAATCCCAACCCAAGAAT 3’

FATP1-F: 5’ AGGTCAATGAGGACACGATGGAG 3’

FATP1-R: 5’ CTGGTACATTGAGTTAGGGTCCAAC 3’

### Statistics

All of the statistical analyses were performed in SPSS version 16 (SPSS Inc., Chicago, IL, USA). The generalized linear model (GLM) procedure was used to analyze the genotype, time, and their interaction as a source of variation. The significance of the differences between all of the groups was analyzed by one-way ANOVA or t-test. A *P* value of <0.05 was considered significant.

## Results

### Tissue dissection

To explore the FA distribution in the whole body and in various tissues from lean and obese mice, each mouse was weighed and dissected into a total of 18 components as described in the materials and methods section. Whole body weights and the weight of each organ/tissue from the lean and obese mice are listed in S1 Table. The obese mice were heavier than the lean mice (*P*<0.05), with an average body weight of 41.05 g at 4h to 46.54 g at 168h for the obese mice compared with 16.07 g at 4h to 17.62 g at 168h for the lean mice. The largest component of all of the tissues/organs collected from lean mice was muscle (18.52%), followed by skin (10.85%), liver (7.01%) and adipose tissue (6.65%). Adipose tissue was the largest component from the obese mice (32.44%), followed by skin (16.76%), liver (10.47%) and muscle (7.42%). The recovery of tissues/organs was calculated as the percentage of the total weight of the collected tissues/organs to the live body weight. There was no difference in tissue/organ recovery between the lean and obese mice (81.89±2.43% versus 85.55±1.41%; n = 18; *P*>0.05).

### Whole body distribution (%) and total ^14^C-label content in various tissues of lean and obese mice

**Fig 1:**
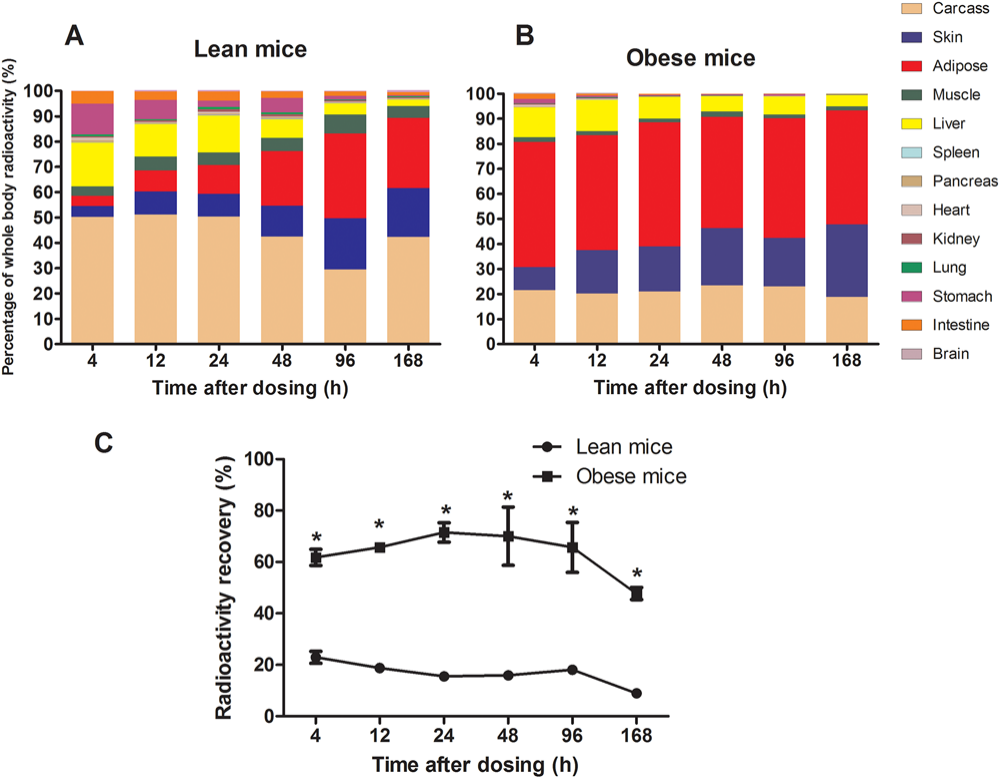
Whole body distribution (%) of ^14^C-label in various organs/tissues from lean mice (A) and obese mice (B). The mean recovery (%) of the oral dose of ^14^C-label at various time points in the whole body of lean and obese mice (C). The total amount of ^14^C-label per organ/tissue was calculated based on the concentration data and the total organ/tissue weight. The total ^14^C-label contents in the tissues and carcasses were summed to calculate the whole body total content and the recovery. The data for each organ/tissue are presented as a percentage of the whole body total radioactivity at each time point, and the values are presented as the mean ± SEM (n = 3). Asterisks (*) indicate significant differences between lean and obese mice at a particular time point (*P*<0.05).

The total amounts of ^14^C-label from the tissues and carcasses were summed to calculate the whole body total radioactivity and the recovery. The data for each tissue from the lean mice are presented as a percentage of the whole body total radioactivity at each time point (Fig 1A, S2 Table). In addition to the carcass, a large amount of ^14^C-label was observed in the liver (17.3%), stomach (12.3%), intestine (4.9%), skin (4.2%), adipose tissue (4.1%) and muscle (3.8%) at 4 h. The amount of ^14^C-label in the gastrointestinal tract decreased rapidly and was barely detectable at 168 h. The ^14^C-label content in skin, adipose tissue and muscle gradually increased and peaked with proportions of 20.3%, 33.4% and 7.5%, respectively, at 96 h. The major reservoir for ^14^C-label at 168 h was adipose tissue (27.84%), and secondary reservoirs included skin (19.29%) and muscle (4.66%). Very little ^14^C-label was detected in other tissues, such as spleen (0.12%), pancreas (0.23%), heart (0.21%), kidney (0.42%), lung (0.31%) and brain (0.46%), at 168 h. The whole body distribution of [1-^14^C] 18:1n-9 in obese mice is shown in Fig 1B (S2 Table). The ^14^C-label was found predominantly in adipose tissue (49.9%), liver (12.0%), skin (9.2%), intestine (2.1%), stomach (1.8%) and muscle (1.8%) at 4 h. The ^14^C-label content in the gastrointestinal tract decreased gradually and was barely detectable at 168 h. Adipose tissue and muscle exhibited constant ^14^C-label content, with proportions of 44.39–49.94% and 1.4–2.2%, respectively, but the proportions of the ^14^C-label in skin markedly increased between 4 h (9.23%) and 168 h (28.86%). The ^14^C-label content in the liver decreased from 12.0% at 4 h to 4.5% at 168 h. The major reservoir for ^14^C-label in obese mice at 168 h was adipose tissue (45.52%), with additional reservoirs in skin (28.86%), liver (4.48%) and muscle (1.62%). Very little ^14^C-label was detected at 168 h in other tissues, such as spleen (0.01%), pancreas (0.08%), heart (0.05%), kidney (0.12%), lung (0.12%), and brain (0.03%), and all of these tissues had considerably less ^14^C-label content in obese mice than in lean mice.

The total amount of ^14^C-label per organ/tissue was calculated from the concentration data and the total organ/tissue weight (Table 1). Two-way ANOVA analysis showed genotype significantly affected the total ^14^C-label contents in skin (*P*<0.001), adipose tissue (*P*<0.001), muscle (*P =* 0.026), liver (*P*<0.001), spleen (*P*<0.001), heart (*P*<0.001), lung (*P =* 0.021), stomach (*P*<0.001), intestine (*P =* 0.022), brain (*P*<0.001) and the carcass (*P*<0.001), whereas the total ^14^C-label contents in pancreas (*P =* 0.996) and kidney (*P =* 0.207) were not significantly affected by genotype. Time exerted significant effect on the total ^14^C-label contents in most of tissues except adipose tissue (*P* = 0.18) and brain (*P* = 0.725). The genotype and time interactions were found in muscle (*P =* 0.026), spleen (*P*<0.001), heart (*P*<0.001), kidney (*P*<0.001), lung (*P*<0.001) and stomach (*P*<0.001), whereas ^14^C-label contents in skin (*P =* 0.163), adipose tissue (*P =* 0.187), liver (*P =* 0.314), pancreas (*P =* 0.056), intestine (*P =* 0.145), brain (*P =* 0.067) and the remainder of the carcass (*P =* 0.138) were not affected by the interaction between genotype and time.

**Table 1:** Total ^14^C-label contents in various tissues of lean and obese mice from 4h to 168h after a single oral dose^1^.

Tissues	Total ^14^C-label contents (dpm, × 10^4^)													*P*-value		
	Lean mice						Obese mice						SEM	Genotype	Time	Genotype × Time
	4h	12h	24h	48h	96h	168h	4h	12h	24h	48h	96h	168h				
Skin	4.5	7.9	6.4	8.9	17.0	8.0	26.4	53.0	59.7	73.9	58.7	63.6	7.95	<0.001***	0.049*	0.163
Adipose tissue^2^	4.3	7.2	8.2	15.9	28	11.5	142.6	139.2	163.5	143.6	145	100.4	12.4	<0.001***	0.18	0.187
Muscle^3^	4.0	4.8	3.6	3.8	6.3	1.9	5.1	4.9	5.0	7.0	4.4	3.6	0.68	0.026*	0.009**	0.026*
Liver	18.4	11.1	10.5	5.6	3.8	1.0	34.3	37.9	28.4	20	22.1	9.9	3.69	<0.001***	<0.001***	0.314
Spleen	0.12	0.11	0.16	0.14	0.09	0.05	0.17	0.1	0.08	0.05	0.05	0.02	0.012	<0.001***	<0.001***	<0.001***
Pancreas	0.7	0.3	0.36	0.37	0.27	0.1	0.52	0.38	0.38	0.26	0.38	0.18	0.059	0.996	<0.001***	0.056
Heart	1.2	0.28	0.48	0.25	0.21	0.09	2.4	1.0	0.27	0.2	0.23	0.11	0.073	<0.001***	<0.001***	<0.001***
Kidney	0.57	0.58	0.78	0.61	0.41	0.17	1.0	0.8	0.6	0.4	0.3	0.25	0.048	0.207	<0.001***	<0.001***
Lung	0.63	0.35	0.59	0.51	0.27	0.13	0.5	0.8	0.16	0.14	0.22	0.27	0.047	0.021*	<0.001***	<0.001***
Stomach	13.0	6.7	1.9	4.2	1.2	0.18	5.0	2.3	1.3	1.33	1.5	0.1	0.90	<0.001***	<0.001***	0.001**
Intestine	5.1	2.9	2.5	1.9	1.5	0.53	5.9	2.1	1.7	0.68	0.56	0.32	0.37	0.022*	<0.001***	0.145
Brain	0.16	0.18	0.21	0.18	0.18	0.19	0.08	0.084	0.06	0.09	0.10	0.07	0.012	<0.001***	0.725	0.067
Carcass^4^	53.4	44.5	36.3	31.2	24.6	17.5	61.6	61.1	69.3	75.9	69.9	41.7	7.79	<0.001***	0.021*	0.138

^1^The data are presented as the mean (n = 3),

*P < 0.05,

**P < 0.01,

***P < 0.001

^2^Adipose: including subcutaneous, visceral, perirenal and mesenteric adipose tissue;

^3^Muscle: all harvested muscles from the arms and legs as well as dorsal muscle;

^4^Carcass: Including the skull, ribs, bones, cartilage, paws, tail and the parts not listed above

The amount of ^14^C-label recovered in the whole body was estimated by adding the quantity of label in individual compartments (S3 Table). The mean recoveries of oral [1-^14^C] 18:1n-9 at 4, 12, 24, 48, 96 and 168 h in lean mice were 22.98±2.34%, 18.83±1.76%, 15.57±0.28%, 15.94±1.71%, 18.12±1.48% and 8.97±0.97%, respectively; these values were significantly lower than those obtained at the same time points in obese mice (61.80±3.16%, 65.72±1.33%, 71.53±3.80%, 70.03±11.36%, 65.68±9.70% and 47.72±2.38%, respectively) (*P*<0.05, Fig 1C). The higher whole body total radioactivity and recovery demonstrated that obese mice retained and stored more FAs within their bodies after dosing than lean mice.

### Time course of ^14^C-label concentration in various tissues from lean and obese mice

**Fig 2:**
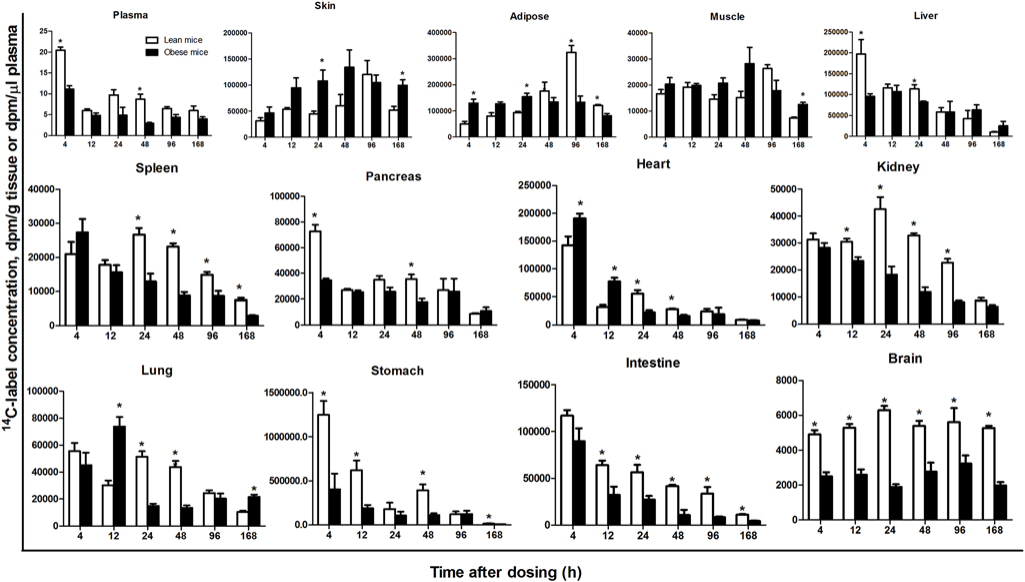
Time course (0–168 h) of the ^14^C-label concentrations (dpm/g tissue and dpm/μl plasma) in plasma, skin, liver, muscle, adipose tissue, spleen, pancreas, heart, kidney, lung, stomach, intestine and brain from lean and obese mice after a single oral dose. The data are presented as the mean ± SEM (n = 3). Asterisks (*) indicate significant differences between lean and obese mice at a particular time point (*P*<0.05). Muscle: all harvested muscles from the arms and legs as well as dorsal muscle. Adipose: subcutaneous, visceral, perirenal and mesenteric adipose tissue.

The time course and individual tissue accumulation of a pulse of FAs were systematically followed to determine the uptake and retention of FAs in various tissues. The appearance of FAs and the maximal FA concentrations in various tissues from lean and obese mice are presented for each time point after a single oral dose of [1-^14^C] 18:1n-9 (Fig 2). The maximum radioactivity of ^14^C-label (dpm/g or dpm/ml) in the plasma, liver, pancreas, heart, lung, stomach and intestine from lean mice occurred at 4 h and decreased thereafter. ^14^C-label radioactivity increased gradually in spleen and kidney from 4 to 24 h after administration and decreased gradually from 24 to 168 h. However, the highest radioactivity concentration peaked at 96 h in several other tissues from lean mice, including skin, adipose tissue and muscle. No obvious ^14^C-label concentration peak was observed in the brain. A different pattern was observed in obese mice. The peak concentrations of ^14^C-label (dpm/g or dpm/ml) in the plasma, liver, spleen, heart, kidney, stomach, and intestine of obese mice occurred at 4 h and decreased thereafter. The ^14^C-label concentration increased in skin and muscle from 4 to 48 h and reached a maximum at 48 h. The ^14^C-label concentration peaked in lung at 12 h and decreased thereafter. No obvious peaks in the ^14^C-label concentration were observed from 4 to 168 h in adipose tissue, pancreas or brain. However, most of the tissues examined in this study showed a decreasing trend at 168 h compared with the earlier time points.

### ^14^C-label concentration in the SOL, GAS and EDL muscles

**Fig 3:**
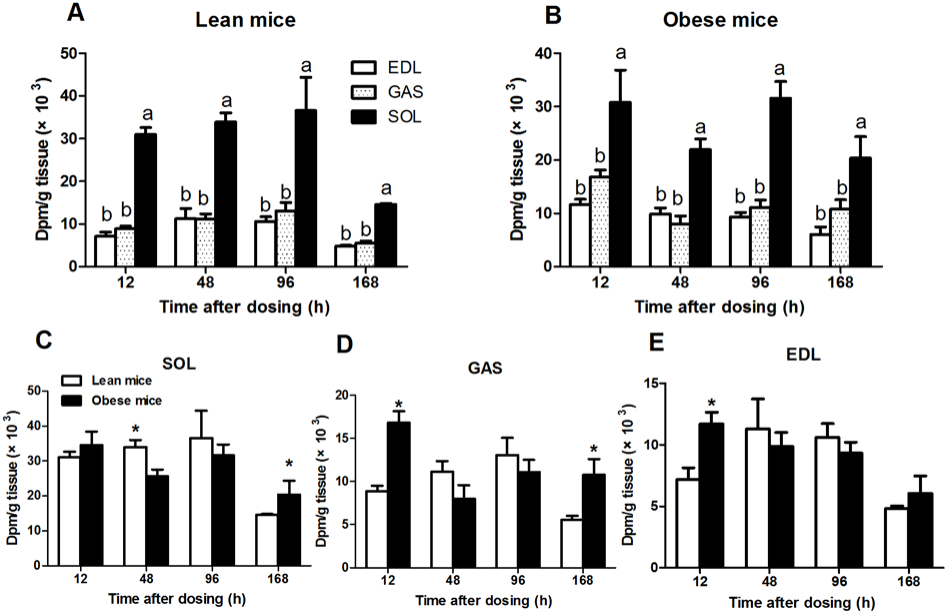
The concentration of ^14^C-label (dpm/g tissue) in the EDL, GAS and SOL from lean mice (A) and obese mice (B). Comparative study of the ^14^C-label concentration (dpm/g tissue) in the EDL (C), GAS (D) and SOL (E) between lean and obese mice. The data are presented as the mean ± SEM (n = 3). Different letters indicate significant differences between different muscle types at a particular time point (*P* < 0.05). Asterisks (*) indicate significant differences between lean and obese mice at a particular time point (*P*<0.05). EDL, extensor digitorum longus; GAS, gastrocnemius; SOL, soleus.

Muscle from obese individuals exhibits alterations in FA metabolism, and these alterations are fiber type-specific [1]. Therefore, three muscles (GAS, EDL and SOL) were collected for radioactivity assays to explore muscle type variations in FA uptake. The ^14^C-label content in various muscles at different time points is presented in Fig 3. Generally, the SOL exhibited a significantly higher ^14^C-label concentration than the GAS and EDL (*P*<0.05), whereas no significant differences were observed in the ^14^C-label content between the GAS and EDL in lean and obese mice (*P*>0.05, Fig 3A and 3B). The ^14^C-label concentration in the SOL, GAS and EDL exhibited an increasing trend from 12 to 96 h and decreased at 168 h in lean mice (Fig 3A). However, the total radioactivity peaked in the SOL, GAS and EDL at 12 h and then displayed a decreasing trend thereafter in obese mice (Fig 3B). The GAS and EDL from obese mice had significantly higher ^14^C-label concentrations compared with lean mice at 12 h (*P*<0.05, Fig 3D and 3E). The ^14^C-label concentrations were significantly higher at 168 h in the SOL and GAS from obese mice compared with lean mice (*P*<0.05, Fig 3C and 3D).

### Gene expression in the SOL, GAS and EDL muscles

**Fig 4:**
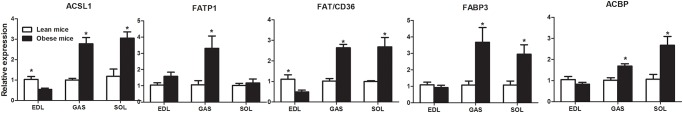
Comparative study of the mRNA expression of ACSL1, FAT, ACBP, FABP3, and FATP1 in the EDL, GAS and SOL between lean and obese mice. The data are presented as the mean ± SEM (n = 3). Asterisks (*) indicate significant differences between lean and obese mice (*P*<0.05). ACSL, long-chain fatty acyl-CoA synthetase; FAT, fatty acid translocase; ACBP, acyl CoA binding protein; FABP, fatty acid binding protein; FATP, fatty acid transport protein, EDL, extensor digitorum longus; GAS, gastrocnemius; SOL, soleus.

Alterations in FA uptake are important for the regulation of FA disposal; therefore, the expression of genes involved in LCFA uptake in muscle was analyzed. The gene expression of ACSL1, FATP1, FAT/CD36, ACBP and FABP3 was highest in the SOL of lean and obese mice (S1 Fig). The SOL and GAS exhibited significantly higher expression levels of ACSL1, FAT/CD36, ACBP and FABP3 in obese mice than in lean mice (*P*<0.05, Fig 4). ACSL1 and FAT/CD36 expression was significantly lower in the EDL from obese mice than from lean mice (*P*<0.05, Fig 4).

### ^14^C-label content in various fat depots in lean and obese mice

**Fig 5:**
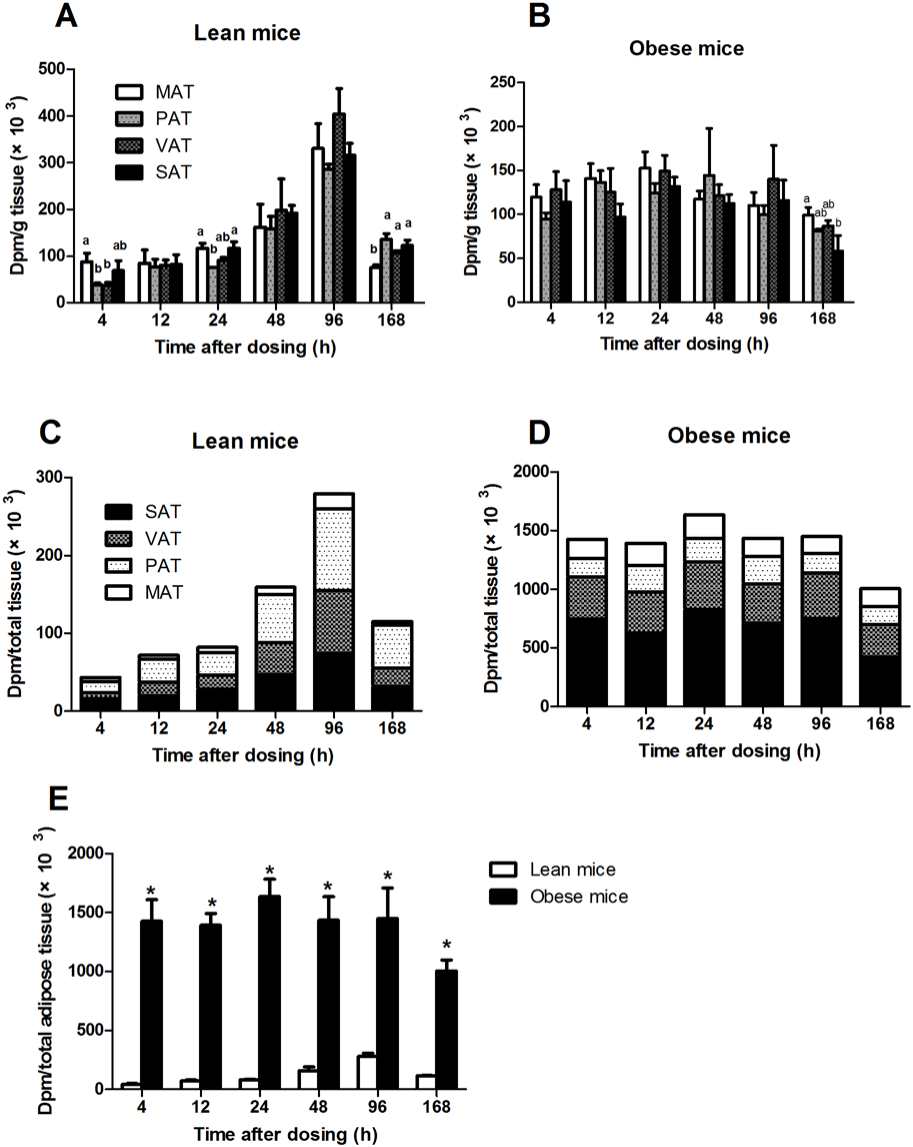
The concentration of ^14^C-label (dpm/g tissue) in various fat depots (MAT, PAT, VAT and SAT) from lean mice (A) and obese mice (B). The total ^14^C-label content (dpm/total tissue) in various fat depots (MAT, PAT, VAT and SAT) from lean mice (C) and obese mice (D). Comparison of the total ^14^C-label content in adipose tissue (MAT+PAT+VAT+SAT) between lean and obese mice (E). The data are presented as the mean ± SEM (n = 3). Different letters indicate significant differences between different adipose depots at a particular time point (*P*<0.05). Asterisks (*) indicate significant differences between lean and obese mice at a particular time point (*P*<0.05). MAT, mesenteric adipose tissue, PAT, perirenal adipose tissue, VAT, visceral adipose tissue, SAT, subcutaneous adipose tissue.

Body fat distribution is an important predictor of the metabolic complications of obesity. Variations in body fat accumulation result from regional differences in FA disposal [21]. Therefore, four fat depots were collected and analyzed using radioactivity assays to explore regional variations in FA uptake and retention. The ^14^C-label concentrations (dpm/g tissue) in the four fat depots at different time points in lean and obese mice are presented in Fig 5. No significant differences were observed in the ^14^C-label concentration among MAT, PAT, VAT and SAT at 12, 48, and 96 h in lean mice (*P*>0.05). There was greater retention in MAT and SAT compared with PAT and VAT at 4 and 24 h, and MAT had the lowest ^14^C-label at 168 h among the four fat depots in lean mice (Fig 5A). No significant differences in ^14^C-label concentration were observed among the four adipose depots at time points prior to 168 h in obese mice (*P*>0.05, Fig 5B). MAT had a significantly higher ^14^C-label concentration at 168 h compared with SAT (*P*<0.05), and PAT and VAT had intermediate concentrations; this pattern differed from that in lean mice (Fig 5B). When the data from 4 to 168 h in lean mice were expressed as ^14^C-label content per total tissue (dpm/total tissue), the ^14^C-label content was highest in PAT, followed by SAT and VAT, and MAT has the lowest content (Fig 5C, S4 Table). However, SAT in obese mice had the highest ^14^C-label content, which was significantly higher than in VAT. The ^14^C-label contents in PAT and MAT were similar in obese mice and were significantly lower than in SAT and VAT (*P*<0.05, Fig 5D, S4 Table). The total ^14^C-label content in the four adipose tissue depots (dpm/total tissue) was significantly higher in obese mice than in lean mice (Fig 5E).

### Gene expression in various fat depots from lean and obese mice

**Fig 6:**
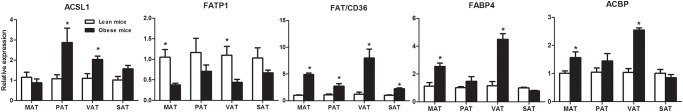
Comparative study of the mRNA levels of ACSL1, FAT, ACBP, FABP3, and FATP1 in MAT, PAT, VAT and SAT between lean and obese mice. The data are presented as the mean ± SEM (n = 3). Asterisks (*) indicate significant differences between lean and obese mice (*P*<0.05). ACSL, long-chain fatty acyl-CoA synthetase; FAT, fatty acid translocase; ACBP, acyl CoA binding protein; FABP, fatty acid binding protein; FATP, fatty acid transport protein.

No significant differences were observed in ACSL1 and FABP3 expression in MAT, PAT, VAT and SAT from lean mice (*P*>0.05, S2 Fig). In lean mice, FAT/CD36 expression was highest in SAT, followed by PAT, and the lowest expression was observed in MAT. ACBP gene expression was higher in MAT and SAT than in PAT and VAT from lean mice (*P*<0.05). FATP1 mRNA expression was highest in VAT and lowest in MAT among the four adipose depots in lean mice (S2 Fig). ACSL1, FAT/CD36, FABP3 and FATP1 gene expression in obese mice was highest in VAT, and the expression of ACSL1, FAT/CD36 and FATP1 was lowest in MAT. ACBP expression was higher in MAT and VAT than in PAT and SAT (*P*<0.05, S2 Fig). FAT/CD36 gene expression was significantly higher in all four fat depots from obese mice compared with lean mice (*P*<0.05, Fig 6). The four fat depots from lean mice showed a trend of higher FATP1 gene expression compared with obese mice, but no significant differences were observed in PAT and SAT expression (*P*>0.05). ACBP and FABP4 displayed similar expression patterns among the four adipose depots, and their expression was significantly higher in MAT and VAT from obese mice than from lean mice (*P*<0.05). ACSL1 was significantly more abundant in PAT and VAT from obese mice than from lean mice (*P*<0.05).

### ^14^C distribution (%) in different lipid classes in SAT from lean and obese mice

SAT had the highest ^14^C-label content among the four fat depots in obese mice. In obese mice, SAT accounted for approximately half of the total ^14^C-label content; this was much higher than the proportion of SAT (approximately 30% of the total) in lean mice. Total lipid extraction and TLC were conducted to explore the differences in ^14^C distribution (%) in various lipid classes in SAT from lean and obese mice (Table 2). More than 80% of the lipid radioactivity was recovered as esterified lipids at each time point. Approximately half of the lipid radioactivity in SAT from obese mice was recovered as TAG, and this proportion of TAG was significantly higher than that in SAT from lean mice (*P*<0.05) at each time point.

**Table 2:** Lipid class distribution (%) of ^14^C-label in SAT from lean and obese mice^12^.

Lipid class	Time after feeding, h			
	4	12	24	168
Lean mice	%			
CE	11.9 ± 0.7^b^	11.0 ± 2.6^b^	14.9 ± 1.6^b^	11.2 ± 2.0^c^
TAG	24.7 ± 4.8^a^	35.7 ± 4.9^a^	33.3 ± 2.4^a^	24.1 ± 4.4^b^
FFA	11.3 ± 2.2^b^	14.6 ± 1.3^b^	17.5 ± 2.8^b^	12.8 ± 3.1^bc^
MAG/DAG	23.6 ± 5.9^a^	15.7 ± 4.1^b^	14.8 ± 1.8^b^	8.5 ± 4.9^c^
PL	28.5 ± 1.8^a^	23.0 ± 4.9^b^	19.6 ± 0.8^b^	43.5 ± 0.7^a^
Obese mice	%			
CE	8.6 ± 0.8^b^	11.8 ± 2.7^c^	12.7 ± 2.3^b^	16.4 ± 4.6^b^
TAG	45.8 ± 6.3^a^	44.2 ± 1.8^a^	44.3 ± 9.3^a^	47.8 ± 2.4^a^
FFA	14.3 ± 3.2^b^	11.6 ± 1.4^c^	16.4 ± 7.2^b^	13.6 ± 2.4^b^
MAG/DAG	13.8 ± 1.1^b^	21.4 ± 2.7^b^	9.5 ± 0.6^b^	14.3 ± 4.5^b^
PL	17.6 ± 6.4^b^	11.1 ± 0.9^c^	17.1 ± 4.1^b^	7.9 ± 0.5^b^

^1^The data shows the percentage of each lipid class in total lipid of SAT from lean or obese mice. Data are presented as the mean±SEM (n = 3). The means in the columns with superscripts without a common letter differ (*P*<0.05).

^2^SAT, subcutaneous adipose tissue; CE, cholesterol ester; TAG, triglycerol; FFA, free fatty acids; MAG/DAG, mono- and diacylglycerol; PL, phospholipid;

## Discussion

To our knowledge, no comparative study has analyzed FA uptake and distribution in all major tissues/organs in lean and obese mammals. This study systematically evaluated the time course and individual tissue/organ accumulation of a pulse of ^14^C-labeled FAs, which is isotopic tracer metabolized in the same manner as natural FFA [22].

Our results show that the majority of the ^14^C-label was found in the carcass (containing the skull, ribs, bones, cartilage, paws, tail and the parts not listed in Fig 1), adipose tissue and skin in both lean and obese mice (Fig 1, S4 Table), which was consistent with the results from other animals, like guinea pig [23] and rat [24,25], in which most LCFAs were deposited in the carcass, adipose tissue and skin. Our data reveal the different patterns of FA uptake and retention in obese mice compared with lean mice. The amount of ^14^C-label in adipose tissue, muscle and skin gradually increased to a peak at 96 h in lean mice, whereas the ^14^C-label accumulation in the carcass, adipose tissue and skin accounted for more than 95% of the whole body distribution at any time point in obese mice and no obvious uptake or storage peak was observed in adipose tissue and muscle between 4 and 168 h. The ^14^C-label content in adipose tissue accounted for more than 44% of the total content in obese mice, which was much higher than that in lean mice (4.1–33.3% of the total from 4 to 168h). This result that obese mice took up and deposited more FAs in adipose tissue than did lean mice indicates that obese mice exhibit a higher rate/capacity for FA uptake and accumulation in adipose tissue, which may contribute to obesity.

FA uptake into tissues affected by time [24] or genotype [1] has been reported previously, but few report was related to the interaction between genotype and time on FA uptake. Here we found that the ^14^C-label content in adipose tissue was only affected by genotype, whereas muscle ^14^C-label content was affected by genotype, time and the interaction between genotype and time. We don’t know the rationale for the difference, but the effect of genotype on FA uptake and retention in adipose and skeletal muscle tissues must underlie the obesity of *ob/ob* mice.

The total ^14^C-labeled retention was 9.0% in lean mice at 168 h, indicating that approximately 91.0% of the label was expired as ^14^CO_2_ or secreted in urine and feces, which is consistent with previous results in rats [18]. In obese mice, 47.7% of the ^14^C-label was retained at 168 h, suggesting that approximately 52.3% was secreted or expired. Total radioactivity in urine and feces was not detected in this study, but a previous study showed that 1.4–3.8% of ^14^C-labeled unsaturated FAs were recovered from urine and feces, which are relatively small proportions compared with the total oxidation. The recovery of labeled FAs reflects their oxidation rates, with greater FA retention indicating less oxidation. Therefore, it can be inferred that there was less oxidation in obese mice than in lean mice, which may be due to decreased oxidation in adipose tissue of obese mice. Our results confirmed previous studies showing a reduction in the metabolic rate [26] and a marked decrease in energy expenditure [27] in *ob/ob* mice.

The peak FA concentration in a tissue/organ partially reflects the capacity of the particular tissue/organ to uptake and deposit FAs. The radioactivity data in Fig 2 demonstrate that adipose tissue, liver, heart and skin have the highest capacity for FA uptake; all of these tissues/organs had a peak radioactivity of greater than 100,000 dpm/g of tissue/organ. Spleen, pancreas, kidney and lung mobilized FAs in lean and obese mice, with radioactivity values in the range of 20,000–80,000 dpm/g tissue. The very low radioactivity in brain indicated that this tissue has a limited capacity for FA uptake and utilization compared with the other tissues/organs analyzed in this study.

The earlier appearance of a radioactivity peak and the higher radioactivity in obese mice (at 4h) compared with lean mice (at 96h) (Fig 2) suggested that obese mice have a greater ability to uptake, accumulate and store FAs in adipose tissue than lean mice. This result was supported by the lower concentration of ^14^C-label in the plasma, stomach and intestine of obese mice. The preference for FA storage in adipose tissue in *ob/ob* mice likely underlies obesity. A higher concentration of ^14^C-label was observed in skeletal muscle from obese mice at 168 h, suggesting that the reduced catabolism of FAs could contribute to obesity. In addition, different concentrations of ^14^C-label were observed between the two mouse genotypes in other organs, including the spleen, pancreas, kidney, lung and brain. The biological significance of the differential mobilization of FAs in these organs is unclear, but it may be a feature of the knockout of leptin in *ob/ob* mice.

The radioactivity in different adipose depots, including MAT, PAT, VAT and SAT, and in different muscles was analyzed in this study to further characterize FA mobilization in these tissues. As expected, the slow oxidative SOL muscle, which primarily uses FAs for energy generation, had the highest concentration of ^14^C in lean and obese mice (Fig 3A and 3B). This result confirmed the observation that the patterns of FA utilization are related to fiber type, with slow-twitch oxidative muscles having a higher capacity for FAs uptake and lipid oxidation than fast-twitch glycolytic muscle fibers [28–30]. The increased radioactivity in GAS and EDL muscles from obese mice suggested that *ob/ob* mice tend to deposit more FAs in skeletal muscle, which contributes to insulin resistance in obesity [1]. The total radioactivity in the four adipose tissue depots revealed that obese mice had greater FA deposition than lean mice (Fig 5E). The opposite FAs uptake/accumulation pattern in MAT and SAT suggested that FA storage was altered in MAT and SAT in obese mice. An emerging consensus is that visceral fat is particularly damaging because it portends a greater risk for diabetes, cardiovascular disease, hypertension, and certain types of cancer [31]. The altered FA distribution/storage among different fat depots in *ob/ob* mice likely contributes to the susceptibility of these mice to these diseases. Although the ^14^C-label concentration in SAT was lower in obese mice, SAT had the highest total ^14^C-label content among the four fat depots. The study of lipid class distribution revealed that significantly more (*P*<0.05) ^14^C-label was recovered as TAG in SAT from obese mice than from lean mice, which likely contributes to the higher fat mass in SAT in obese mice.

The expression patterns of ACBP in lean mice and of ACBP and FABP3 in obese mice were consistent with the uptake pattern of the ^14^C-label in various fat depots at the first time point (4 h). The mRNA expression of most genes, with the exception of FATP1, was higher in obese mice; the FATP1 mRNA levels decreased in various adipose depots in obese mice, confirming the observations by Memon [11]. FATP1 has been proposed to play a role in the delivery of FAs to mitochondria, and down-regulated FATP expression may decrease the flow of FAs toward mitochondria for β-oxidation [11,32]. Higher expression of these genes was observed in the SOL, which corresponds with the uptake and accumulation of FAs in the three different skeletal muscles. Taken together, our results suggested that ACBP and FABP3 may play important roles in FA uptake in adipose tissue, at least at earlier time points. ACSL1, FAT, ACBP, FATP1 and FABP3 play important and unique roles in LCFA uptake in muscle compared with adipose tissue.

## Conclusions

The current study revealed that the pattern of FA uptake and distribution in various tissues and organs was altered in obese mice, and these alterations were tissue-specific. Oxidative catabolism was lower in obese mice than in lean mice, mainly due to decreased oxidation in adipose tissue. Obese mice tend to deposit more FAs in skeletal muscle compared with lean mice. ACBP and FABP3 may play important roles in FA uptake in adipose tissue, and ACSL1, FAT, ACBP, FATP1 and FABP3 play important roles in LCFA uptake in skeletal muscle. The altered functions in FA mobilization in *ob/ob* mice may contribute to obesity.

## Supporting Information

S1 Fig The mRNA levels of ACSL1, FAT, ACBP, FABP3, and FATP1 in the EDL, GAS and SOL from lean mice (A) and obese mice (B). The data are presented as the mean ± SEM (n = 3). Different letters indicate significant differences between different muscle types (*P*<0.05). ACSL, long-chain fatty acyl-CoA synthetase; FAT, fatty acid translocase; ACBP, acyl CoA binding protein; FABP, fatty acid binding protein; FATP, fatty acid transport protein, EDL, extensor digitorum longus; GAS, gastrocnemius; SOL, soleus.
(TIF)

S2 Fig The mRNA levels of ACSL1, FAT, ACBP, FABP3, and FATP1 in mesenteric (MAT), perirenal (PAT), visceral (VAT) and subcutaneous (SAT) adipose tissue from lean mice (A) and obese mice (B). The data are presented as the mean ± SEM (n = 3). Different letters indicate significant differences between different adipose depots (*P*<0.05). ACSL, long-chain fatty acyl-CoA synthetase; FAT, fatty acid translocase; ACBP, acyl CoA binding protein; FABP, fatty acid binding protein; FATP, fatty acid transport protein.
(TIF)

S1 Table Body weight and the weight of each organ/tissue. (DOCX)

S2 Table Whole body distribution (%) of ^14^C-label in various organs/tissues from lean and obese mice. (DOCX)

S3 Table Total ^14^C-label retention in the entire body of lean and obese mice. (DOCX)

S4 Table Percentage of ^14^C-label retention in each adipose tissue depot from lean and obese mice. (DOCX)

## References

[1] Turcotte LP, Swenberger JR, Zavitz Tucker M, Yee AJ. Increased fatty acid uptake and altered fatty acid metabolism in insulin-resistant muscle of obese Zucker rats. Diabetes 2001;50: 1389–1396. 11375340 10.2337/diabetes.50.6.1389

[2] Faergeman NJ, Knudsen J. Role of long-chain fatty acyl-CoA esters in the regulation of metabolism and in cell signalling. Biochem J. 1997;323 (Pt 1): 1–12. 9173866 10.1042/bj3230001 PMC1218279

[3] Masuzaki H, Paterson J, Shinyama H, Morton NM, Mullins JJ, Seckl JR, et al A transgenic model of visceral obesity and the metabolic syndrome. Science 2001;294: 2166–2170. 11739957 10.1126/science.1066285

[4] Rinaldo P. Fatty acid transport and mitochondrial oxidation disorders. Semin Liver Dis. 2001;21: 489–500. 11745037 10.1055/s-2001-19037

[5] Turcotte LP, Swenberger JR, Tucker MZ, Yee AJ, Trump G, Luiken JJ, et al Muscle palmitate uptake and binding are saturable and inhibited by antibodies to FABP(PM). Mol Cell Biochem. 2000;210: 53–63. 10976758 10.1023/a:1007046929776

[6] Svedberg J, Bjorntorp P, Smith U, Lonnroth P. Free-fatty acid inhibition of insulin binding, degradation, and action in isolated rat hepatocytes. Diabetes 1990;39: 570–574. 2185108 10.2337/diab.39.5.570

[7] Arner P. Catecholamine-induced lipolysis in obesity. Int J Obes Relat Metab Disord. 1999;23 Suppl 1: 10–13. 10193856 10.1038/sj.ijo.0800789

[8] Macotela Y, Emanuelli B, Mori MA, Gesta S, Schulz TJ, Tseng YH, et al Intrinsic differences in adipocyte precursor cells from different white fat depots. Diabetes 2012; 61: 1691–1699. 10.2337/db11-1753 22596050 PMC3379665

[9] Goodpaster BH, Theriault R, Watkins SC, Kelley DE. Intramuscular lipid content is increased in obesity and decreased by weight loss. Metabolism 2000;49: 467–472. 10778870 10.1016/s0026-0495(00)80010-4

[10] Bonen A, Luiken JJ, Liu S, Dyck DJ, Kiens B, Kristiansen S, et al Palmitate transport and fatty acid transporters in red and white muscles. Am J Physiol. 1998;275: E471–478. 9725814 10.1152/ajpendo.1998.275.3.E471

[11] Memon RA, Fuller J, Moser AH, Smith PJ, Grunfeld C, Feingold KR. Regulation of putative fatty acid transporters and Acyl-CoA synthetase in liver and adipose tissue in ob/ob mice. Diabetes 1999;48: 121–127. 9892232 10.2337/diabetes.48.1.121

[12] Coburn CT, Hajri T, Ibrahimi A, Abumrad NA. Role of CD36 in membrane transport and utilization of long-chain fatty acids by different tissues. J Mol Neurosci. 2001;16: 117–121. 11478366 10.1385/JMN:16:2-3:117

[13] Glatz JF, Luiken JJ, van Bilsen M, van der Vusse GJ. Cellular lipid binding proteins as facilitators and regulators of lipid metabolism. Mol Cell Biochem. 2002;239: 3–7. 12479562

[14] Huang H, Starodub O, McIntosh A, Kier AB, Schroeder F. Liver fatty acid-binding protein targets fatty acids to the nucleus. Real time confocal and multiphoton fluorescence imaging in living cells. J Biol Chem. 2002;277: 29139–29151. 12023965 10.1074/jbc.M202923200

[15] Chiu HC, Kovacs A, Ford DA, Hsu FF, Garcia R, Herrero P, et al A novel mouse model of lipotoxic cardiomyopathy. J Clin Invest. 2001;107: 813–822. 11285300 10.1172/JCI10947 PMC199569

[16] Knudsen J. Acyl-CoA-binding protein (ACBP) and its relation to fatty acid-binding protein (FABP): an overview. Mol Cell Biochem. 1990;98: 217–223. 2266962 10.1007/BF00231387

[17] Stahl A. A current review of fatty acid transport proteins (SLC27). Pflugers Arch. 2004;447: 722–727. 12856180 10.1007/s00424-003-1106-z

[18] Leyton J, Drury PJ, Crawford MA. Differential oxidation of saturated and unsaturated fatty acids in vivo in the rat. Br J Nutr. 1987;57: 383–393. 3109464 10.1079/bjn19870046

[19] Folch J, Lees M, Sloane Stanley GH. A simple method for the isolation and purification of total lipides from animal tissues. J Biol Chem. 1957;226: 497–509. 13428781

[20] Wang XX, Huang M, Wang YZ. The Effect of Insulin, TNF alpha and DHA on the Proliferation, Differentiation and Lipolysis of Preadipocytes Isolated from Large Yellow Croaker (Pseudosciaena Crocea R.). Plos One 2012;7: e48069 10.1371/journal.pone.0048069 23110176 PMC3482209

[21] Jensen MD, Sarr MG, Dumesic DA, Southorn PA, Levine JA. Regional uptake of meal fatty acids in humans. Am J Physiol Endocrinol Metab. 2003;285: E1282–1288. 12915396 10.1152/ajpendo.00220.2003

[22] Koutsari C, Dumesic DA, Patterson BW, Votruba SB, Jensen MD. Plasma free fatty acid storage in subcutaneous and visceral adipose tissue in postabsorptive women. Diabetes 2008;57: 1186–1194. 10.2337/db07-0664 18285557

[23] Fu Z, Attar-Bashi NM, Sinclair AJ. 1-14C-linoleic acid distribution in various tissue lipids of guinea pigs following an oral dose. Lipids 2001;36: 255–260. 11337980 10.1007/s11745-001-0715-7

[24] Lin YH, Salem N Jr. Whole body distribution of deuterated linoleic and alpha-linolenic acids and their metabolites in the rat. J Lipid Res. 2007;48: 2709–2724. 17876057 10.1194/jlr.M700369-JLR200

[25] Cunnane SC. The Canadian Society for Nutritional Sciences 1995 Young Scientist Award Lecture. Recent studies on the synthesis, beta-oxidation, and deficiency of linoleate and alpha-linolenate: are essential fatty acids more aptly named indispensable or conditionally dispensable fatty acids? Can J Physiol Pharmacol. 1996;74: 629–639. 8909772

[26] Breslow MJ, Min-Lee K, Brown DR, Chacko VP, Palmer D, Berkowitz DE. Effect of leptin deficiency on metabolic rate in ob/ob mice. Am J Physiol. 1999;276: E443–449. 10070008 10.1152/ajpendo.1999.276.3.E443

[27] Pelleymounter MA, Cullen MJ, Baker MB, Hecht R, Winters D, Boom T, et al Effects of the obese gene product on body weight regulation in ob/ob mice. Science 1995;269: 540–543. 7624776 10.1126/science.7624776

[28] Budohoski L, Gorski J, Nazar K, Kaciuba-Uscilko H, Terjung RL. Triacylglycerol synthesis in the different skeletal muscle fiber sections of the rat. Am J Physiol. 1996;271: E574–581. 8843753 10.1152/ajpendo.1996.271.3.E574

[29] Dyck DJ, Peters SJ, Glatz J, Gorski J, Keizer H, Kiens B, et al Functional differences in lipid metabolism in resting skeletal muscle of various fiber types. Am J Physiol.1997;272: E340–351. 9124537 10.1152/ajpendo.1997.272.3.E340

[30] Pagliassotti MJ, Pan D, Prach P, Koppenhafer T, Storlien L, et al Tissue oxidative capacity, fuel stores and skeletal muscle fatty acid composition in obesity-prone and obesity-resistant rats. Obes Res.1995;3: 459–464. 8521165 10.1002/j.1550-8528.1995.tb00175.x

[31] Bergman RN, Kim SP, Catalano KJ, Hsu IR, Chiu JD, et al Why visceral fat is bad: Mechanisms of the metabolic syndrome. Obesity 2006;14: 16s–19s. 16642958 10.1038/oby.2006.277

[32] Martin G, Schoonjans K, Lefebvre AM, Staels B, Auwerx J. Coordinate regulation of the expression of the fatty acid transport protein and acyl-CoA synthetase genes by PPARalpha and PPARgamma activators. J Biol Chem. 1997;272: 28210–28217. 9353271 10.1074/jbc.272.45.28210

