# Supplementary material for: Alterations in Oral [1-14C] 18:1n-9 Distribution in Lean Wild-Type and Genetically Obese (ob/ob) Mice
Source: PLoS One. 2015 Mar 31;10(3):e0122028. doi: 10.1371/journal.pone.0122028 (PMC4380473; doi:10.1371/journal.pone.0122028)
Supplement: S1 Table — (DOCX) [file pone.0122028.s003.docx]

| Tissues | Tissue Weight (g, at various time points) | | | | | | | | | | | | | | | | | | | | | |
| --- | --- | --- | --- | --- | --- | --- | --- | --- | --- | --- | --- | --- | --- | --- | --- | --- | --- | --- | --- | --- | --- | --- |
|  | 0 | |  | 4 | |  | 12 | | |  | 24 | |  | 48 | |  | 96 | | |  | 168 | |
|  | Lean | Obese |  | Lean | Obese |  | Lean | Obese | |  | Lean | Obese |  | Lean | Obese |  | Lean | | Obese |  | Lean | Obese |
| Skin | 1.42±0.04 | 5.55±0.10 |  | 1.43±0.04 | 5.69±0.09* |  | 1.48±0.05 | | 5.63±0.11* |  | 1.44±0.02 | 5.50±0.08* |  | 1.46±0.02 | 5.53±0.14* |  | | 1.40±0.03 | 5.63±0.20* |  | 1.55±0.04 | 6.40±0.09* |
| Adipose | 0.87±0.02 | 10.74±0.19 |  | 0.88±0.02 | 11.01±0.17* |  | 0.91±0.03 | | 10.89±0.22* |  | 0.88 ±0.01 | 10.64±0.15* |  | 0.90±0.01 | 10.71±0.28* |  | | 0.86±0.02 | 10.90±0.38* |  | 0.95±0.03 | 12.38±0.17* |
| Muscle | 2.41±0.06 | 2.46±0.04 |  | 2.44±0.07 | 2.52±0.04 |  | 2.52±0.08 | | 2.49±0.05 |  | 2.45±0.03 | 2.43±0.03 |  | 2.50±0.03 | 2.45±0.06 |  | | 2.39±0.05 | 2.49± 0.09 |  | 2.64±0.08 | 2.83±0.04 |
| Liver | 0.91±0.02 | 3.47±0.06 |  | 0.92±0.03 | 3.55±0.05* |  | 0.96±0.03 | | 3.52±0.07* |  | 0.93±0.01 | 3.43±0.05* |  | 0.95±0.01 | 3.46±0.09* |  | | 0.90±0.02 | 3.52±0.12* |  | 1.00±0.03 | 4.00±0.06* |
| Stomach | 0.10±0.00 | 0.12±0.00 |  | 0.11±0.00 | 0.12±0.00* |  | 0.11±0.00 | | 0.12±0.00* |  | 0.11±0.00 | 0.12±0.00* |  | 0.11±0.00 | 0.12±0.00* |  | | 0.10±0.00 | 0.12±0.00* |  | 0.11±0.00 | 0.14±0.00* |
| Intestine | 0.44±0.01 | 0.64±0.01 |  | 0.44±0.01 | 0.66±0.01* |  | 0.46±0.01 | | 0.65±0.01* |  | 0.44±0.01 | 0.63±0.01* |  | 0.45±0.01 | 0.64±0.02* |  | | 0.43±0.01 | 0.65±0.02* |  | 0.48±0.01 | 0.74±0.01* |
| Heart | 0.09±0.00 | 0.12±0.00 |  | 0.09±0.00 | 0.12±0.00* |  | 0.09±0.00 | | 0.12±0.00* |  | 0.09±0.00 | 0.12±0.00* |  | 0.09±0.00 | 0.12±0.00* |  | | 0.08±0.00 | 0.12±0.00* |  | 0.09±0.00 | 0.14±0.00* |
| Pancreas | 0.10±0.00 | 0.15±0.00 |  | 0.10±0.00 | 0.15±0.00* |  | 0.10±0.00 | | 0.15±0.00* |  | 0.10±0.00 | 0.15±0.00* |  | 0.10±0.00 | 0.15±0.00* |  | | 0.10±0.00 | 0.15±0.01* |  | 0.11±0.00 | 0.17±0.00* |
| Kidney | 0.18±0.00 | 0.34±0.01 |  | 0.18±0.01 | 0.35±0.01* |  | 0.19±0.01 | | 0.34±0.01* |  | 0.18±0.00 | 0.34±0.00* |  | 0.19±0.00 | 0.34±0.01* |  | | 0.18±0.00 | 0.34±0.01* |  | 0.20±0.01 | 0.39±0.01* |
| Lung | 0.11±0.00 | 0.11±0.00 |  | 0.11±0.00 | 0.11±0.00 |  | 0.12±0.00 | | 0.11±0.00 |  | 0.11±0.00 | 0.11±0.00 |  | 0.12±0.00 | 0.11±0.00* |  | | 0.11±0.00 | 0.11±0.00 |  | 0.12±0.00 | 0.12±0.00 |
| Spleen | 0.06±0.00 | 0.06±0.00 |  | 0.06±0.00 | 0.06±0.00 |  | 0.06±0.00 | | 0.06±0.00 |  | 0.06±0.00 | 0.06±0.00 |  | 0.06±0.00 | 0.06±0.00 |  | | 0.06±0.00 | 0.06±0.00 |  | 0.06±0.00 | 0.07±0.00* |
| Brain | 0.33±0.01 | 0.32±0.01 |  | 0.33±0.01 | 0.33±0.01 |  | 0.34±0.01 | | 0.32±0.01 |  | 0.33±0.00 | 0.32±0.00* |  | 0.34±0.00 | 0.32±0.01* |  | | 0.32±0.01 | 0.32±0.01 |  | 0.36±0.01 | 0.37±0.01 |
| Carcass | 6.03±0.16 | 9.11±0.13 |  | 6.08±0.17 | 9.11±0.13* |  | 6.30±0.20 | | 9.30±0.19* |  | 6.12±0.07 | 9.05±0.09* |  | 6.24±0.08 | 9.34±0.28* |  | | 5.96±0.12 | 9.31±0.25* |  | 6.60±0.19 | 9.70±0.22* |
| Body weight (g) | 16.07±0.31 | 41.05±0.46* |  | 16.04±0.09 | 41.39±0.64* |  | 16.71±0.22 | | 40.94±0.81* |  | 16.04±0.17 | 39.99±0.56* |  | 16.39±0.23 | 40.25±1.05* |  | | 15.81±0.52 | 40.98±1.44* |  | 17.62±0.58 | 46.54±0.64* |
| Total tissues weight (g) | 13.04±0.35 | 34.50±0.33* |  | 13.17±0.36 | 35.14±0.53* |  | 13.62±0.44 | | 35.04±0.70* |  | 13.25±0.15 | 34.20±0.45* |  | 13.50±0.16 | 34.64±0.86* |  | | 12.91±0.26 | 35.08±1.15* |  | 14.28±0.41 | 38.96±0.19* |
| Recovery (%) | 81.16±1.18 | 84.09±1.36 |  | 82.09±1.88 | 84.89±0.06 |  | 81.52±1.76 | | 85.58±0.08 |  | 82.63±1.47 | 85.51±0.09 |  | 82.38±1.60 | 86.66±0.00 |  | | 81.69±1.23 | 85.60±0.19* |  | 81.06±1.11 | 83.74±0.77 |

S1 Table The weight of the dissected organ/tissues from lean and obese mice*

*Data are presented as Mean ± SEM (n=3). Total tissues weight: Total weight of all collected tissues/organs. Recovery: (tissue weight/body weight)×100%.
