# Supplementary material for: Alterations in Oral [1-14C] 18:1n-9 Distribution in Lean Wild-Type and Genetically Obese (ob/ob) Mice
Source: PLoS One. 2015 Mar 31;10(3):e0122028. doi: 10.1371/journal.pone.0122028 (PMC4380473; doi:10.1371/journal.pone.0122028)
Supplement: S2 Table — (DOCX) [file pone.0122028.s004.docx]

S2 Table Whole body distribution (%) of [1-^14^C] 18:1n-9 in various organs/tissues from lean and obese mice^1^

| % of whole body total radioactivity (at various time points) | | | | | | | | | | | | | |
| --- | --- | --- | --- | --- | --- | --- | --- | --- | --- | --- | --- | --- | --- |
| Tissues | lean mice | | | | | |  | Obese mice | | | | | |
|  | 4h | 12h | 24h | 48h | 96h | 168h |  | 4h | 12h | 24h | 48h | 96h | 168h |
| Skin | 4.20 | 9.12 | 8.94 | 12.15 | 20.29 | 19.29 |  | 9.23 | 17.45 | 18.08 | 22.85 | 19.34 | 28.86 |
| Adipose | 4.06 | 8.28 | 11.41 | 21.64 | 33.37 | 27.84 |  | 49.94 | 45.84 | 49.49 | 44.39 | 47.80 | 45.52 |
| Muscle | 3.79 | 5.54 | 4.93 | 5.11 | 7.51 | 4.66 |  | 1.79 | 1.63 | 1.53 | 2.15 | 1.45 | 1.62 |
| Liver | 17.32 | 12.83 | 14.64 | 7.54 | 4.50 | 2.52 |  | 12.01 | 12.48 | 8.58 | 6.17 | 7.28 | 4.48 |
| Spleen | 0.12 | 0.13 | 0.22 | 0.19 | 0.10 | 0.12 |  | 0.06 | 0.03 | 0.02 | 0.02 | 0.02 | 0.01 |
| Pancreas | 0.69 | 0.32 | 0.49 | 0.50 | 0.32 | 0.23 |  | 0.18 | 0.12 | 0.11 | 0.08 | 0.13 | 0.08 |
| Heart | 1.15 | 0.33 | 0.67 | 0.34 | 0.25 | 0.21 |  | 0.83 | 0.31 | 0.08 | 0.06 | 0.08 | 0.05 |
| Kidney | 0.54 | 0.66 | 1.08 | 0.83 | 0.48 | 0.42 |  | 0.34 | 0.26 | 0.18 | 0.12 | 0.09 | 0.12 |
| Lung | 0.59 | 0.41 | 0.82 | 0.69 | 0.33 | 0.31 |  | 0.17 | 0.26 | 0.05 | 0.04 | 0.07 | 0.12 |
| Stomach | 12.28 | 7.65 | 2.61 | 5.73 | 1.46 | 0.43 |  | 1.76 | 0.75 | 0.38 | 0.41 | 0.50 | 0.05 |
| Intestine | 4.85 | 3.35 | 3.48 | 2.56 | 1.73 | 1.27 |  | 2.07 | 0.69 | 0.52 | 0.21 | 0.18 | 0.15 |
| Brain | 0.15 | 0.21 | 0.29 | 0.25 | 0.21 | 0.46 |  | 0.03 | 0.03 | 0.02 | 0.03 | 0.03 | 0.03 |
| Carcass^2^ | 50.26 | 51.18 | 50.42 | 42.46 | 29.43 | 42.25 |  | 21.58 | 20.14 | 20.96 | 23.47 | 23.03 | 18.91 |

^1^The data for each organ/tissue are presented as a percentage of the whole body total radioactivity at each time point, and the values are presented as the mean (n=3).

^2^Carcass：Including the skull, ribs, bones, cartilage, paws, tail and the parts not listed above
