# Supplementary material for: Alterations in Oral [1-14C] 18:1n-9 Distribution in Lean Wild-Type and Genetically Obese (ob/ob) Mice
Source: PLoS One. 2015 Mar 31;10(3):e0122028. doi: 10.1371/journal.pone.0122028 (PMC4380473; doi:10.1371/journal.pone.0122028)
Supplement: S3 Table — (DOCX) [file pone.0122028.s005.docx]

**S3 Table Total ^14^C-label retention in the entire body of lean and obese mice**

|  | Total ^14^C-label retention (dpm, ×10^5^) | | | | | |
| --- | --- | --- | --- | --- | --- | --- |
| Time | 4h | 12 h | 24h | 48h | 96h | 168h |
| Lean mice | 10.6 ± 1.1 | 8.7 ± 0.8 | 7.2 ± 0.1 | 7.7 ± 0.8 | 8.4 ± 0.7 | 4.1 ± 0.4 |
| Obese mice | 28.5 ± 1.5 | 30.4 ± 0.6 | 33.1 ± 1.8 | 32.4 ± 5.2 | 30.3 ± 4.5 | 22.1 ± 1.1 |
| *P* | 0.000593 | 2.91E-05 | 0.000125 | 0.009244 | 0.00836 | 0.000113 |

Data are presented as Mean ± SEM (n=3).
