# Supplementary material for: Alterations in Oral [1-14C] 18:1n-9 Distribution in Lean Wild-Type and Genetically Obese (ob/ob) Mice
Source: PLoS One. 2015 Mar 31;10(3):e0122028. doi: 10.1371/journal.pone.0122028 (PMC4380473; doi:10.1371/journal.pone.0122028)
Supplement: S4 Table — (DOCX) [file pone.0122028.s006.docx]

S4 Table Percentage of ^14^C-label retention in each adipose depot from lean and obese mice

| Tissue | 4 | |  | 12 | |  | 24 | |  | 48 | |  | 96 | |  | 168 | |  |
| --- | --- | --- | --- | --- | --- | --- | --- | --- | --- | --- | --- | --- | --- | --- | --- | --- | --- | --- |
|  | Lean | Obese |  | Lean | Obese |  | Lean | Obese |  | Lean | Obese |  | Lean | Obese |  | Lean | Obese |  |
| SAT | 36.1 ± 5.0^a^ | 51.2 ± 5.7^a*^ |  | 27.6 ± 3.1^b^ | 44.5 ± 4.7^a*^ |  | 34.1 ± 2.6^a^ | 50.7 ± 1.2^a*^ |  | 30.9 ± 3.9^ab^ | 50.1 ± 3.4^a*^ |  | 26.7 ± 0.6^b^ | 51.0 ± 3.9^a*^ |  | 27.8 ± 2.8^b^ | 40.3 ± 9.9^a^ |  |
| VAT | 17.7 ± 1.1^b^ | 25.9 ± 4.5^b^ |  | 24.7 ± 5.5^b^ | 25.3 ± 5.9^b^ |  | 22.5 ± 0.9^b^ | 24.8 ± 0.9^b^ |  | 24.5 ± 3.6^b^ | 23.5 ± 1.3^b^ |  | 28.4 ± 1.5^b^ | 26.6 ± 3.2^b^ |  | 20.3 ± 1.2^c^ | 28.6 ± 5.3^b^ |  |
| PAT | 34.1 ± 3.6^a*^ | 11.6 ± 1.6^c^ |  | 40.9 ± 2.4^a*^ | 16.4 ± 2.0^b^ |  | 35.0 ± 2.1^a*^ | 12.4 ± 0.3^c^ |  | 38.7 ± 1.1^a*^ | 15.5 ± 3.7^bc^ |  | 38.1 ± 1.8^a*^ | 11.8 ± 1.1^c^ |  | 47.7 ± 3.6^a*^ | 15.6 ± 2.0^b^ |  |
| MAT | 12.2 ± 1.2^b^ | 11.4 ± 0.1^c*^ |  | 6.8 ± 1.2^c^ | 13.7 ± 2.1^b*^ |  | 8.4 ± 0.5^c^ | 12.1 ± 0.5^c*^ |  | 5.9 ± 0.7^c^ | 10.9 ± 0.9^c*^ |  | 6.8 ± 0.7^c^ | 10.6 ± 1.7^c^ |  | 4.2 ± 0.4^d^ | 15.4 ± 2.7^b*^ |  |

Data are presented as Mean ± SEM (n=3). Different letters indicates significant difference in same column at *P*<0.05. [Asterisk](javascript:void(0);) (*) indicates significant differences between lean and obese mice at that time point at *P*< 0.05. SAT: subcutaneous adipose tissue; VAT: visceral adipose tissue; PAT: perirenal adipose tissue; MAT: Mesenteric adipose tissue
